# Reduced vocal variability in a zebra finch model of dopamine depletion: implications for Parkinson disease

**DOI:** 10.14814/phy2.12599

**Published:** 2015-11-12

**Authors:** Julie E Miller, George W Hafzalla, Zachary D Burkett, Cynthia M Fox, Stephanie A White

**Affiliations:** 1 Departments of Neuroscience and Speech, Language and Hearing Sciences of the University of ArizonaTucson, Arizona; 2 Integrative Biology & Physiology, University of CaliforniaLos Angeles, California; 3 National Center for Voice and SpeechDenver, Colorado

**Keywords:** 6-hydroxydopamine, Parkinson disease, songbird, zebra finch

## Abstract

Midbrain dopamine (DA) modulates the activity of basal ganglia circuitry important for motor control in a variety of species. In songbirds, DA underlies motivational behavior including reproductive drive and is implicated as a gatekeeper for neural activity governing vocal variability. In the zebra finch, *Taeniopygia guttata*, DA levels increase in Area X, a song-dedicated subregion of the basal ganglia, when a male bird sings his courtship song to a female (female-directed; FD). Levels remain stable when he sings a less stereotyped version that is not directed toward a conspecific (undirected; UD). Here, we used a mild dose of the neurotoxin 6-hydroxydopamine (6-OHDA) to reduce presynaptic DA input to Area X and characterized the effects on FD and UD behaviors. Immunoblots were used to quantify levels of tyrosine hydroxylase (TH) as a biomarker for DA afferent loss in vehicle- and 6-OHDA-injected birds. Following 6-OHDA administration, TH signals were lower in Area X but not in an adjacent subregion, ventral striatal-pallidum (VSP). A postsynaptic marker of DA signaling was unchanged in both regions. These observations suggest that effects were specific to presynaptic afferents of vocal basal ganglia. Concurrently, vocal variability was reduced during UD but not FD song. Similar decreases in vocal variability are observed in patients with Parkinson disease (PD), but the link to DA loss is not well-understood. The 6-OHDA songbird model offers a unique opportunity to further examine how DA loss in cortico-basal ganglia pathways affects vocal control.

## Introduction

**Figure 1:**
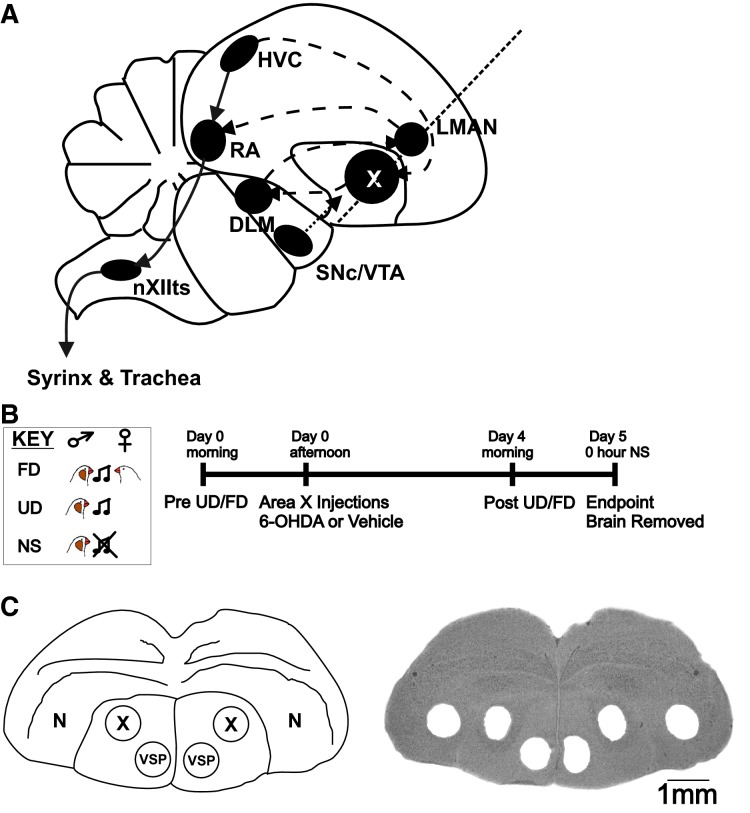
Neuroanatomy of the song circuitry and experimental timeline. (A) The song control system mainly consists of two interconnected loops: the vocal production pathway (solid lines) containing cortical nuclei HVC (proper name) and the robust nucleus of the arcopallium (RA); the anterior forebrain pathway (dashed lines) including basal ganglia Area X (X), the dorsolateral division of the medial thalamus (DLM) and the cortical lateral magnocellular nucleus of the anterior nidopallium (LMAN). Area X receives DA input (dotted arrow) from the substantia nigra pars compacta (SNc) and ventral tegmental area (VTA) (Gale and Perkel 2010). A dotted line indicates the coronal plane of section shown for (C). Modified from (Miller et al. 2008). nXIIts –tracheosyringeal portion of the hypoglossal motor nucleus. Other abbreviations in text. (B) Experimental timeline. The key (Fig. 1B), represents the behavioral contexts for 2 h of undirected (UD) song, female directed (FD) song and 0 h non-singing (NS), the experimental endpoint. In the case of insufficient singing, this timeline was adjusted ±1 day for pre-surgery and post-surgery song collection. (C) Schematic of male zebra finch coronal brain section (left) indicates anatomical regions and micropunches in the thionin-stained section (right).

Songbirds offer attractive models for investigation of brain–behavior relationships at gene, circuit, and organismal levels. They share similar reciprocally connected cortico-striatal loops with mammals, but offer the additional advantage of a well-characterized neural circuitry for vocalization. Critically, the loci for song production are neuroanatomically distinct and well-characterized including Area X, the specialized subregion of the songbird basal ganglia dedicated to vocal learning and maintenance (Fig.1A). As in mammals, the basal ganglia receive dopaminergic innervation from the midbrain ventral tegmental area (VTA) and substantia nigra pars compacta (SNc) (Bottjer 1993; Lewis et al. 1981). Feedback loops exist between these regions and Area X, as in mammalian striatum (Person et al. 2008; Gale and Perkel 2010).

Dopamine (DA) orchestrates a delicate balance within mammalian and songbird basal ganglia circuits in processes associated with motor exploration versus performance and reward-based behavior (Shiflett and Balleine 2011). The songbird model enables further exploration of the role of DA in these processes, given that it contains medium spiny neurons (MSNs) and globus-pallidal neurons in the basal ganglia, both of which are DA-sensitive (Simonyan et al. 2012). Both cell types share similar anatomical and physiological signatures with their mammalian counterparts (Farries and Perkel 2002; Goldberg and Fee 2010). Levels of DA in the songbird basal ganglia are closely associated with social and breeding contexts. For example, in Area X of male European starlings and zebra finches, DA levels increase in the presence of a conspecific female, underlying his motivation to sing, as assessed through measurements of the rate-limiting catecholamine biosynthetic enzyme, tyrosine hydroxylase (TH) (Heimovics and Riters 2008) and microdialysis of DA metabolites (Sasaki et al. 2006). In contrast to the female-directed (FD) song, DA levels are lower during undirected (UD) song, when the male sings alone (Hara et al. 2007). DA cells in the VTA provide one source of neuromodulation onto MSNs to regulate these social-context-dependent singing behaviors (Yanagihara and Hessler 2006). Targeting these Area X inputs using neurotoxins can yield insight into the neuromodulation of vocal behavior.

In rodents, injecting the neurotoxin 6-hydroxydopamine (6-OHDA) into either the medial forebrain bundle or striatum poisons DA nerve terminals measured by TH immunostaining. This results in motor phenotypes characteristic of Parkinson disease (PD) with altered ultrasonic vocalizations detected at 72 h and 4 weeks post-injection (Grant et al. 2015). In zebra finches, a unilateral injection of 6-OHDA into the VTA/SNc reduces TH immunostaining in Area X (Hara et al. 2007). Consequently, FD song becomes slower. No overt changes in motif structure were noted, but acoustic variations in individual syllables were not assessed.

Here, 6-OHDA was injected directly into Area X to assess consequences on DA biomarkers and song. We hypothesized that 6-OHDA administration to Area X would reduce DA signal during both UD and FD song and lead to changes in song features. We also predicted that UD would be more sensitive to DA depletion given that levels are relatively low under this condition (Hara et al. 2007); further reduction could drain any reservoir of signal. In normal adult males, higher levels of DA during FD song (Sasaki et al. 2006) are associated with less vocal variability but when D1 receptors are blocked, FD song resembles the more variable UD song (Leblois et al. 2010). Similarly, we predicted that with experimental DA depletion following 6-OHDA injection, acoustic features of FD song would resemble UD. To measure changes in DA signal in the basal ganglia, TH and dopamine receptor-associated postsynaptic protein (DARPP-32) levels were quantified using an approach, not feasible in rodent models, of separately micro-punching vocal versus non-vocal subregions followed by immunoblotting for DA biomarkers. Below, evidence is provided for depletion of presynaptic DA terminals specifically within vocal Area X and associated changes to UD, but not FD, song. No apparent effect of experimental DA depletion in the social context was detected.

## Methods

### Subjects

All animal use was approved by the Institutional Animal Care and Use Committee at the Universities of California Los Angeles and Arizona. For the experiments 25 birds were used, including a subset for tissue analyses. Adult male zebra finches (120–400 days) were moved to individual sound attenuation chambers and acclimated under a 13:11 h light:dark cycle. Behavioral experiments were conducted in the morning from lights-on until overdose with inhalation anesthetic. Brain tissue for immunoblotting and immunohistochemistry was collected from birds that were euthanized immediately following lights-on (0 h nonsinging, NS, Fig.1B) to prevent any confound of behavioral context and/or circadian changes.

### Behavior

Methods followed those of Miller et al. (2008) with some time-course modifications (Fig.1B). UD song recording was ongoing. FD song was captured on the day prior to or the morning of surgery and on day 4 or 5 post-surgery, depending on when singing levels were sufficient. Non-vocal behavior was simultaneously video-taped pre and post-treatment during song recording. An experimenter blind to the treatment scored the pre versus post-surgery occurrence of non-song behaviors during 30 min of UD and FD song beginning at lights-on.

### Song recording and analysis

Sounds were recorded (Shure SM58/93 microphones) and digitized (PreSonus Firepod/Audiobox: 44.1 kHz sampling rate/24 bit depth). Recordings were managed using Sound Analysis Pro (SAP) (Tchernichovski et al. 2000).

The song was hand-segmented following Miller et al. (2010). Motifs were identified as a repeated order of multiple syllables, excluding introductory notes and unlearned calls. Syllables were identified as sound envelopes that could be separated from other syllables by local minima. WAV files from 20 consecutive renditions of motifs and syllables during UD and FD songs were selected (Audacity, audacityteam.org) from a similar morning time-point and run in SAP for measures of self-accuracy and individual acoustic features (SAP manual). The mean with standard error (SEM), and coefficient of variation (CV) of each feature are reported in Table1 with power analysis reported for each acoustic feature. Syllable types known as harmonic stacks were analyzed separately for changes in fundamental frequency (FF) variability (Kao et al. 2005) using code provided by M. Brainard, UCSF (MATLAB, Mathworks, Natick, MA).

**Table 1:** Summary of song features

	6-OHDA												Vehicle											
	Pre		Post		Stats		Pre		Post		Stats		Pre		Post		Stats		Pre		Post		Stats	
Feature	Mean	SEM	Mean	SEM	*P*-value	Power	CV	SEM	CV	SEM	*P*-value	Power	Mean	SEM	Mean	SEM	*P*-value	Power	CV	SEM	CV	SEM	*P*-value	Power
UD																								
Similarity	97.781	0.204	97.971	0.156	0.128	0.425	–	–	–	–	–	–	97.849	0.228	98.047	0.217	0.100	0.432	–	–	–	–	–	–
Accuracy	93.746	0.259	94.077	0.207	**0.015**	0.726	–	–	–	–	–	–	94.033	0.186	94.181	0.184	0.298	0.243	–	–	–	–	–	–
Duration	112.927	9.741	115.678	9.914	**<0.001**	0.897	0.032	0.003	0.030	0.003	0.499	0.186	122.790	7.792	124.070	7.967	0.278	0.290	0.042	0.004	0.043	0.004	0.847	0.097
Pitch	879.757	82.384	867.609	72.699	0.556	0.122	0.162	0.033	0.190	0.055	0.519	0.277	1187.255	164.738	1131.548	163.828	0.376	0.151	0.160	0.027	0.127	0.022	0.142	0.364
Pitch goodness	365.952	32.247	354.946	29.771	0.057	0.455	0.096	0.007	0.089	0.006	0.057	0.601	341.068	24.869	341.500	25.536	0.877	0.077	0.093	0.005	0.097	0.006	0.431	0.201
Wiener entropy	2.595	0.125	2.552	0.119	0.103	0.405	0.074	0.004	0.060	0.003	**<0.001**	0.999	2.430	0.098	2.392	0.097	0.253	0.246	0.072	0.004	0.074	0.004	0.548	0.154
Frequency modulation	37.789	1.774	38.945	1.706	**0.006**	0.790	0.092	0.008	0.083	0.006	0.050	0.542	38.916	1.389	38.633	1.385	0.200	0.219	0.080	0.004	0.081	0.005	0.675	0.139
Mean frequency	3617.879	113.442	3666.710	112.739	0.060	0.568	0.065	0.006	0.054	0.005	0.052	0.589	3398.027	101.422	3423.937	101.758	0.437	0.170	0.055	0.005	0.060	0.004	0.345	0.256
FD																								
Similarity	98.113	0.149	98.048	0.167	0.612	0.360	–	–	–	–	–	–	98.275	0.188	98.248	0.151	0.877	0.104	–	–	–	–	–	–
Accuracy	94.599	0.231	94.435	0.244	0.274	0.643	–	–	–	–	–	–	94.850	0.170	94.591	0.147	0.060	0.434	–	–	–	–	–	–
Duration	111.987	10.581	112.510	10.584	0.284	0.269	0.032	0.003	0.030	0.002	0.330	0.242	119.211	9.596	119.470	9.538	0.859	0.083	0.053	0.006	0.050	0.005	0.330	0.270
Pitch	892.068	83.891	903.281	82.723	0.834	0.118	0.168	0.053	0.175	0.038	0.882	0.159	1093.499	186.007	1142.788	185.375	0.448	0.197	0.126	0.032	0.113	0.024	0.708	0.165
Pitch goodness	368.920	33.240	356.644	31.878	**0.021**	0.776	0.089	0.006	0.089	0.006	0.875	0.116	318.414	25.076	314.156	24.651	0.443	0.213	0.099	0.008	0.094	0.007	0.419	0.160
Wiener entropy	2.594	0.126	2.632	0.120	**0.043**	0.527	0.062	0.004	0.057	0.005	0.160	0.305	2.405	0.109	2.476	0.110	**0.006**	0.838	0.061	0.004	0.063	0.005	0.570	0.161
Frequency modulation	36.975	1.806	36.755	1.787	0.586	0.149	0.082	0.007	0.085	0.007	0.550	0.126	37.344	1.530	36.797	1.631	0.213	0.255	0.081	0.004	0.084	0.005	0.449	0.202
Mean frequency	3563.837	135.539	3526.234	134.621	0.364	0.232	0.065	0.007	0.058	0.009	0.275	0.265	3349.944	112.649	3300.613	110.570	0.160	0.285	0.057	0.005	0.054	0.004	0.449	0.187

Flat harmonic syllables only																								
	6-OHDA												Vehicle											
	Mean		Mean		Stats		CV		CV		Stats		Mean		Mean		Stats		CV		CV		Stats	
Feature	Pre	SEM	Post	SEM	*P*-value	Power	Pre	SEM	Post	SEM	*P*-value	Power	Pre	SEM	Post	SEM	*P*-value	Power	Pre	SEM	Post	SEM	*P*-value	Power
UD																								
Pitch	666.560	55.991	667.833	57.149	0.495	0.158	0.010	0.001	0.010	0.001	0.853	0.105	658.195	45.908	660.046	46.065	0.097	0.536	0.012	0.002	0.013	0.001	0.702	0.284
FD																								
Pitch	577.875	12.858	580.019	13.801	0.840	0.117	0.006	0.001	0.007	0.001	0.263	0.261	718.981	64.003	715.870	62.239	0.253	0.319	0.008	0.002	0.009	0.002	0.159	0.418

Mean, standard error (SEM), *P*-values, and power analyses are reported for pre versus post-surgery comparisons for the 6-OHDA and vehicle-injected bird groups for undirected (UD) and female-directed (FD) song. Significance scores are highlighted by their *P*-values obtained from resampling paired-tests on individual syllables pre versus post-surgery. Highlighted (in bold) *P*-values are shown for mean accuracy, syllable duration, FM, and decreased Wiener entropy CV for UD song post-6-OHDA injection.

### Statistics and data presentation

For the means and CVs of all syllable acoustic features and self-similarity scores, resampling one-way ANOVA indicated a significant (*P* < 0.05) within-bird syllable effect. Thus, syllables were treated as independent of each other (Figure S1 and text). No appreciable increase in power was observed in any statistical test when conducted on an *n* > 20 syllables in a given behavioral condition (Miller et al. 2010). Therefore, all analyses were conducted on the first 20 syllable renditions/session.

Song features are presented in Figures 4–7C as effect sizes. Effect size was calculated using the formula (A − B)/(A + B), where A and B are measures of a given feature in two different conditions, respectively (e.g., before vs. after surgery or in FD vs. UD song). For the effects of surgery (Figs. 4–6A), negative values indicate that a given metric is greater before injection of the drug or vehicle whereas positive values indicate the opposite. Values near zero indicate no surgical effect. For the effect of social context (Figs. 6B and 7C), negative bars indicate the value is greater in UD. “Pre” denotes all pre-surgery syllables regardless of the treatment that the bird received. “Vehicle” are comparisons between post-vehicle FD and post-vehicle UD and “6-OHDA” are comparisons between post-6-OHDA FD and post-6-OHDA UD.

Effect size calculations enable statistical comparison *across* treatment groups or social contexts (i.e., 6-OHDA vs. vehicle-injected birds; UD versus FD), which is not possible using the traditional pre versus post-paired plot comparisons done *within* a treatment group. Because this calculation normalizes the scores for each measure, it is not influenced by between-syllable differences. It also allows a single number to represent the effect of a condition on a syllable feature. An unpaired resampling test on the median for each measure evaluated one condition’s effect size against another’s and is reported in the text. Since the effect size calculation places all data on a scale of −1 to 1, we also report all raw untransformed means and SEM in Table1 with *P*-values generated from the raw scores. A significant effect of 6-OHDA injection was assessed when a *P*-value <0.05 was attained for the 6-OHDA group and not in the vehicle group.

Figures S2–S3 show the more conventional paired plots of the coefficient of variation (CV) of fundamental frequency, presented similarly to Kao et al. (2005). For example, song feature scores were collected for a group of syllables before (pre) and after (post) surgical delivery of vehicle or 6-OHDA. All syllables from animals injected with vehicle were then viewed as pairs of pre-vehicle and post-vehicle data, and the statistical significance of the change resulting from vehicle injection was assessed. A *P*-value is determined by resampling the data to repeatedly simulate the null hypothesis in order to see how likely a difference of the magnitude observed would occur by chance. A similar calculation was performed for the syllables of all animals injected with 6-OHDA. A significant effect of 6-OHDA injection was determined by observing a statistically significant difference in the 6-OHDA data pairs that was not present in the vehicle data pairs.

For DA biomarkers, a resampling unpaired difference test was used to detect group differences and confirmed by a Mann–Whitney *U* test. Resampling was also used to assess the treatment effect (vehicle vs. 6-OHDA) on non-song behavior. For a complete description of the use of the resampling method for birdsong analysis and related citations, refer to Miller et al. (2010) and Burkett et al. (2015).

### Surgical procedure and drug dosages

**Figure 2:**
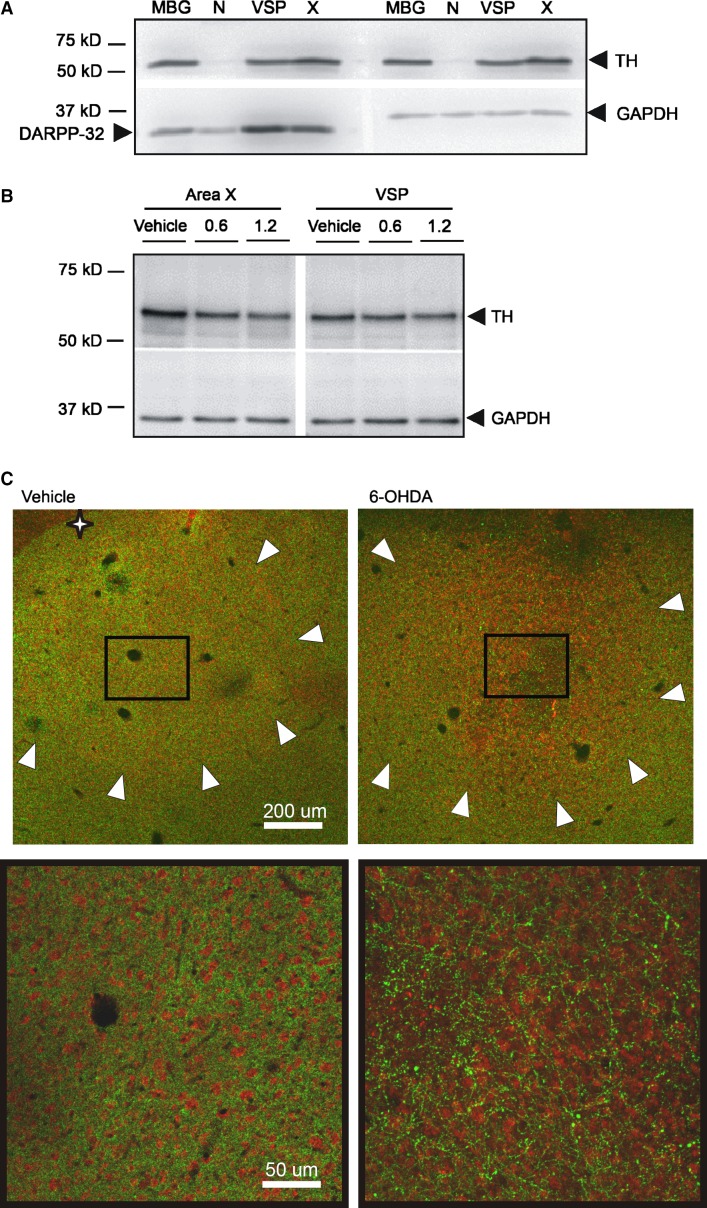
Tissue measurements of DA biomarkers. (A) Immunoblot (40 *μ*g protein/lane) from Area X, VSP, nidopallium (N), and mouse basal ganglia (MBG) lysates. Signals are at the expected molecular weights (kD) and show the expected reduction in TH signal within nidopallium. (B) Immunoblot (15 *μ*g protein/lane) from Area X and VSP lysates. Compared to vehicle (lane 1: 2.82, normalized protein levels), TH signal appeared reduced in Area X following both 0.6 *μ*g (lane 2: 1.75) and 1.2 *μ*g (lane 3: 1.03) doses of 6-OHDA, with more substantial reduction at the higher dose. TH signals in VSP exhibited less change as expected given the targeted injection to Area X (lanes 4–6; vehicle: 1.31; 0.6 *μ*g: 1.06, 1.2 *μ*g: 0.93). (C) Decreased TH immunostaining in Area X following 6-OHDA injection. Photomicrographs show double-labeling for TH positive fibers in green and NeuN, a neuronal marker, in red. The star indicates the striato-pallidal border – beyond this border, the nidopallium lacks the density of TH fibers. Arrowheads outline Area X (top); rectangle highlights inset shown below at higher magnification. There are fewer TH fibers (green) in the 6-OHDA injected Area X compared to vehicle-injected but the density of NeuN staining (red) indicates that Area X neurons are preserved.

Surgery was conducted on isoflurane-anesthetized birds (*n* = 11, bilaterally-injected vehicle birds; *n* = 9, 6-OHDA birds; *n* = 3, received unilateral injections of 6-OHDA and vehicle within the same bird). A glass pipette was fitted into a Nanoject II pressure-injector and back-filled with mineral oil then loaded with either 0.1% sodium L-ascorbate in ddH_2_0 (Sigma #A7631, vehicle) or 6-OHDA (Sigma, lots #MKBPO832V, #MKBR6609V) dissolved in vehicle. A 1.2 *μ*g bilateral dose of 6-OHDA reliably spared the integrity of Area X while leading to subtle effects on song (see Results). Pilot work on the optimal dose to elicit changes in song while preserving Area X integrity indicated that variability in potency across lots of 6-OHDA necessitates testing of each new lot. In pilot work, a higher dose of 4 *μ*g resulted in the same changes in UD song (Miller et al. 2009) reported in this current study at the 1.2 *μ*g dose. However, following this work, a range of 2–4 *μ*g doses of 6-OHDA induced a lesion in Area X that was not due to electrode damage because Area X of vehicle-injected birds remained intact. Studies of rodent models of 6-OHDA have also reported these deleterious effects, using a 6–8 *μ*g dose (M. Ciucci, pers. comm. 2010-2011). Given these considerations, each new vial of drug was tested. Lower doses of 6-OHDA (0.6, 0.8 *μ*g) failed to yield detectable changes in song (data not shown) even though reduced TH levels were observed in immunoblots with as little as 0.6 *μ*g (Fig.2B).

To prevent oxidation, 6-OHDA was prepared within 30 min of use and kept covered on ice to minimize light exposure. Area X was targeted from the bifurcation of the mid-sagittal sinus, in mm: 5.15 rostral, 1.5–1.6 lateral, and a depth of 3.0–3.3. Injections were delivered every 15 sec. The total volume was 250 nL for 6-OHDA (*n* = 9) and 250 or 500 nL for vehicle (*n* = 11). No effect of vehicle volume on song features was observed, so vehicle-injected birds were pooled. After 5 min, the pipette was slowly retracted and the tip visually inspected for clogging. Following post-operative monitoring, birds were returned to their chambers and recorded until death.

### Tissue preparation and immunoblotting

**Figure 3:**
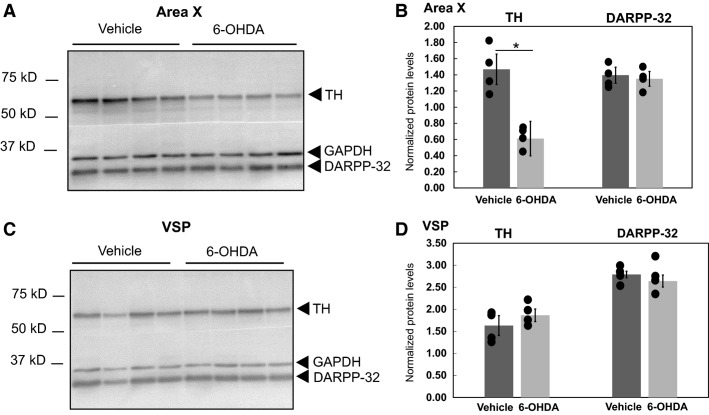
6-OHDA reduces TH signal in Area X but not VSP. (A–B) Immunoblot (A, left; 15 *μ*g/lane) shows decreased TH signal in 6-OHDA- versus vehicle-injected birds, as quantified in the accompanying graph (B, right). No change in DARPP-32 signal was detected. Quantification shows means (bars), standard error (plungers), and individual bird values (circles). (C–D) Immunoblot (C, left; 15 *μ*g/lane) with accompanying graph (D, right) indicates no change in TH and DARPP-32 signals in the VSP for the same birds as in A–B.

Bilateral micropunches of Area X and outlying VSP and nidopallium (N; Fig.1C) were obtained, processed and immunoblotted according to Miller et al. (2008) but with a PVDF membrane. Post hoc thionin staining of punched sections enabled verification of their anatomical precision (Fig.1C). DA biomarkers (Figs.3) were detected with overnight incubation at 4°C with primary antibodies against TH (Millipore #AB152, rabbit 1:500, 1:1500 and DARPP-32 Abcam #ab40801, rabbit 1:10,000, 1:30,000 dilution; Murugan et al. 2013). A primary antibody to GAPDH (Millipore #MAB374, mouse 1:10,000) served as a loading control because neither the Area X protein nor mRNA levels are affected by this behavioral protocol (Miller et al. 2008; Hilliard et al. 2012a). Following TBST washes, blots were probed with HRP secondary antibodies: anti-rabbit IgG (1:2000 – TH, 1:10,000 – DARPP-32) and anti-mouse IgG (1:6000–1:10,000 – GAPDH; Amersham Pharmacia Biotech) for 2 h at room temperature then washed. Blots were developed using chemiluminescence and imaged (Typhoon scanner, or Bio-Rad system) with quantification done in Quantity One (Bio-Rad) by an experimenter blind to the behavioral condition. Densitometric analysis of bands on the immunoblots was as previously described (Miller et al. 2008; Hilliard et al. 2012a). Briefly, a rectangular band was drawn to encapsulate the signal of interest deemed a “raw” value (the “volumetric” measurement in Quantity One) and a same-size rectangular band was placed in the lane above or below the band to subtract the “background” signal. This yielded a corrected value. Corrected values were obtained for TH, DARPP-32 and GAPDH. Corrected values for TH and DARPP-32 were then divided by a corrected GAPDH value per lane to control for equal protein loading. Protein values reported in Figures2–3 represent these normalized values. Results were independently confirmed, using NIH Image J and the same procedure above was based upon densitometric measurements of the bands.

#### Immunohistochemistry

Three adult male zebra finches were injected with 1.2 *μ*g of 6-OHDA in Area X of one hemisphere and with vehicle in the other. Brains were collected following a transcardial perfusion of warmed saline followed by 4% room temperature paraformaldehyde in Phosphate Buffer Saline (PBS) on the morning of day 5 (0HR NS). Fixed brains were cryoprotected in 20% sucrose overnight, then frozen in dry ice and sectioned at 30 *μ*m on a cryostat (Microm). The targeting of Area X was visually verified while sectioning by identification of the electrode track. The injection of 6-OHDA results in a brown discoloration in Area X that is visible to the naked eye. Within a given bird, TH immunostaining was compared between the vehicle-injected versus 6-OHDA injected side. The tissue was double-labeled with TH and the neuronal marker NeuN to confirm that Area X neurons were preserved despite poisoning TH nerve terminals.

Tissue sections were processed as follows: Hydrophobic borders were drawn on the slides, using a pap pen (ImmEdge, Vector Labs) followed by 3 × 5 min washes in TBS with 0.3% Triton X (Tx). To block non-specific antibody binding, the tissue was then incubated for 1 h at room temperature with 5% goat serum in TBS/0.3% Tx then 3 × 5 min washes in 1% goat serum in TBS/0.3% Tx were performed. Primary antibodies to TH (Millipore rabbit 1:500), NeuN (Millipore #MAB377, mouse 1:500) were incubated in a solution of 1% goat serum in TBS/0.3% Tx overnight at 4°C. A “no primary antibody” control was included. The next day, 5 × 5 min washes in TBS/0.3% Tx were performed and sections were incubated for 4 h at room temperature in fluorescently labeled secondary antibodies (Molecular Probes/Life Technologies, 1:1000, goat anti-rabbit 488 #A11034; goat anti-mouse 546 #A11031). Following incubation, 5 × 5 min washes were performed in TBS with filtered TBS used in the last two washes. Slides were then coverslipped in ProLong Anti-Fade Gold mounting medium (Molecular Probes, #P36930), viewed on a confocal microscope (Zeiss LSM 510) using Zeiss LSM software and analyzed, using Adobe Photoshop. In Adobe Photoshop, mean intensity values for TH fiber staining were obtained by measuring the same size rectangular area within Area X of both hemispheres. Mean values for 6-OHDA were then divided by vehicle values to obtain a percentage of TH fiber loss.

## Results

### Validation of DA biomarkers

A polyclonal antibody made against TH (498 aa; GenBank: AAA42258.1) from rat pheochromocytoma was used for detection. This antibody detects TH depletion in rat basal ganglia following 6-OHDA injection into the medial forebrain bundle (Ciucci et al. 2013). The immunizing peptide shares 76% identity to the predicted 491 amino acids in zebra finch TH (∼55 kD; GenBank: XP_002198967). In immunoblots, a robust signal was observed at similar molecular weights across multiple basal ganglia subregions in finch and mouse tissues (Fig.2A). Signals were substantially reduced in the finch nidopallium, consistent with the reduced dopaminergic innervation to this area relative to the basal ganglia in intact birds (Gale and Perkel 2005). A polyclonal antibody against DARPP-32, previously used in zebra finches (Murugan et al. 2013), detects protein signal at the expected molecular weight in region-specific areas of both species (∼32 kD; Fig.2A).

Immunoblots revealed that bilateral injection of either 0.6 *μ*g or 1.2 *μ*g of 6-OHDA into Area X reduced TH levels, with a more pronounced effect at the 1.2 *μ*g dose (Fig.2B). Additionally, fluorescent immunohistochemistry was conducted on fixed coronal tissue sections from birds receiving a unilateral dose of 6-OHDA injected in Area X of one hemisphere and vehicle in the other. In a representative section, intact, densely packed TH positive fibers were detected throughout Area X in the vehicle-injected hemisphere compared with decreased TH fiber staining (by ∼30%) in Area X in the 6-OHDA injected hemisphere (Fig.2C). NeuN staining confirmed that only afferent fibers were lost as the neuronal cell bodies were still present in the 6-OHDA injected Area X (Fig.2C).

### 6-OHDA administration into Area X reduces levels of TH but not DARPP-32 protein

A bilateral injection of 1.2 *μ*g of 6-OHDA into Area X significantly reduced TH signal relative to signals in vehicle-injected birds (Fig.3A and B; *n* = 4/group; mean ± SE: vehicle 1.47 ± 0.19 vs. 6-OHDA 0.61 ± 0.21; resampling mean difference *P* = 0.006). In the outlying VSP from these same birds, no such reduction was observed (Fig.3C and D, mean ± SE: vehicle 1.63 ± 0.22 vs. 6-OHDA 1.90 ± 0.14; *P* = 0.22) indicating that the neurotoxin was confined to Area X. DARPP-32 levels were unaffected in either region (Fig.3, Area X: *P* = 0.56; VSP: *P* = 0.87), suggesting that the 1.2 *μ*g dose damages presynaptic terminals without affecting at least one postsynaptic marker nor inducing MSN cell death, consistent with the NeuN staining described above.

### 6-OHDA administration into Area X decreases vocal variability during UD but not FD song

**Figure 4:**
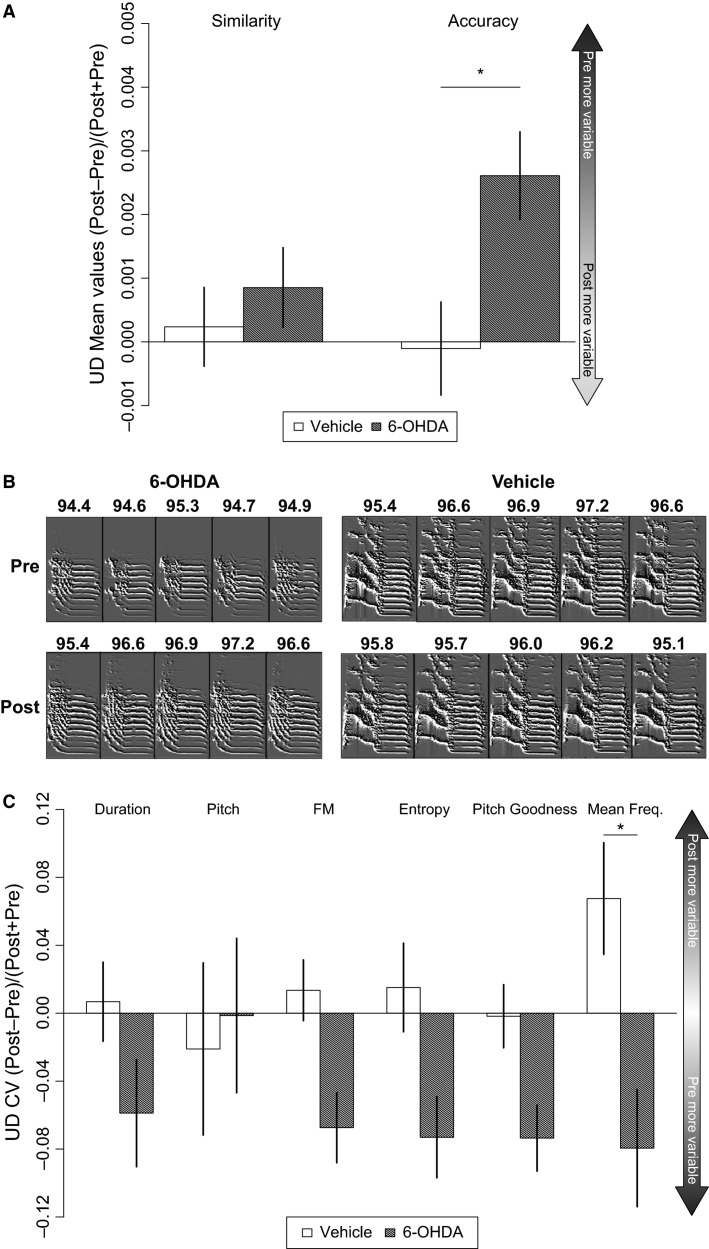
UD features become less variable with 6-OHDA injection in Area X. Effect sizes for the 6-OHDA versus the vehicle condition display the median score with standard error bars from all syllables for each bird. (A) Mean syllable self-accuracy, denoted by the higher bar, is significantly greater post-6-OHDA injection (*) compared to vehicle-injected birds. Self-similarity is not affected by treatment. (B) Consecutive renditions of the same syllable pre versus post-6-OHDA injection or vehicle with mean accuracy scores. Following 6-OHDA injection, the syllable increases in self-accuracy over multiple renditions, not observed in vehicle-injected birds. (C) Duration, frequency modulation (FM), entropy, and pitch goodness show a trend for decreased variability in the post-6-OHDA injected birds compared to vehicle-injected controls. Variability in mean frequency (coefficient of variation, CV) is significantly less (*) following injection of 6-OHDA versus vehicle.

UD song was compared pre and post-bilateral injection of a 1.2 *μ*g dose of 6-OHDA (*n* = 7 birds) or vehicle (*n* = 11 birds) into Area X. Compared to vehicle controls, the 6-OHDA injected birds displayed decreased vocal variability in several acoustic features within the bird’s song, reflected by increased mean accuracy scores for individual syllables when comparing effect sizes (Fig.4A, resampling independent mean differences, *P* = 0.0298) and reflected in the raw mean scores pre versus postsurgery within the 6-OHDA group (Table1). Syllable exemplars also illustrate the increased accuracy (i.e., stereotypy) post-6-OHDA injection (Fig.4B). Reduced variability (CV) in mean frequency was noted for the effect size plots (Fig.4C, resampling independent mean differences, *P* = 0.019). Trends for reduced variability in syllable duration, frequency modulation (FM), entropy, and pitch goodness were also observed in the effect size analysis and met significance for these first three measures in the raw data (Table1).

**Figure 5:**
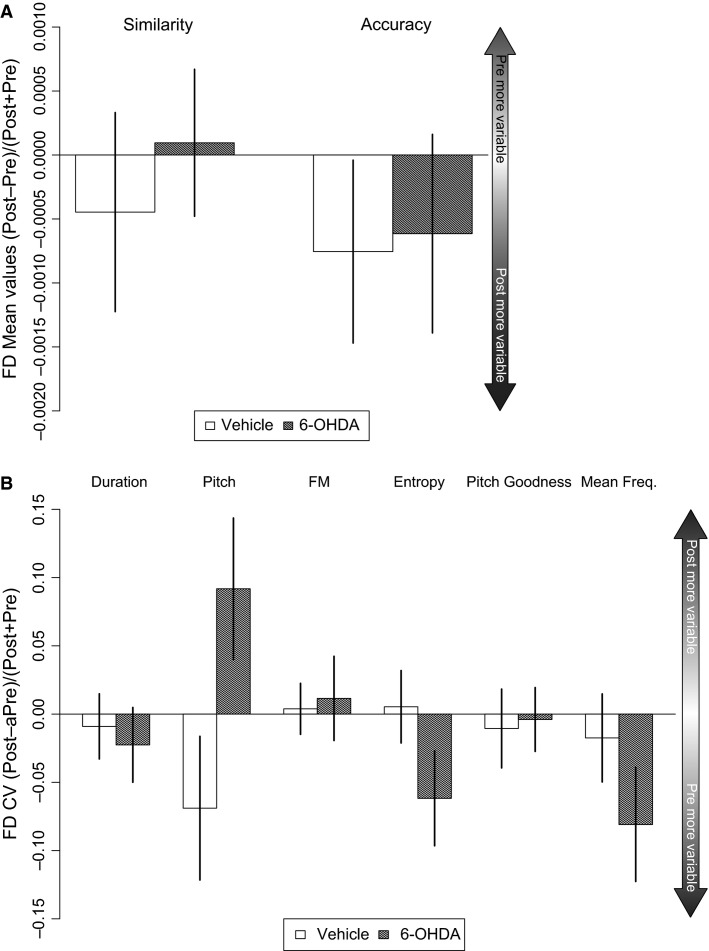
FD features are not affected by 6-OHDA injection in Area X. Effect sizes for each treatment represent the median score with standard error bars from all syllables for each bird in vehicle or 6-OHDA-injected groups. Mean (A) and CV scores (B) in FD song were not significantly affected by 6-OHDA injection.

In these same birds, using the effect size comparison, no significant effects of 6-OHDA were detected on mean and CV scores for syllable features in FD song (Fig.5A and B). An evaluation of the raw scores for pre versus post-6-OHDA injection during FD song, revealed significance for mean pitch goodness that was not present in the vehicle-injected birds (Table1, *P* = 0.021, resampling paired differences).

### No effects of 6-OHDA on social-context-dependent song differences

**Figure 6:**
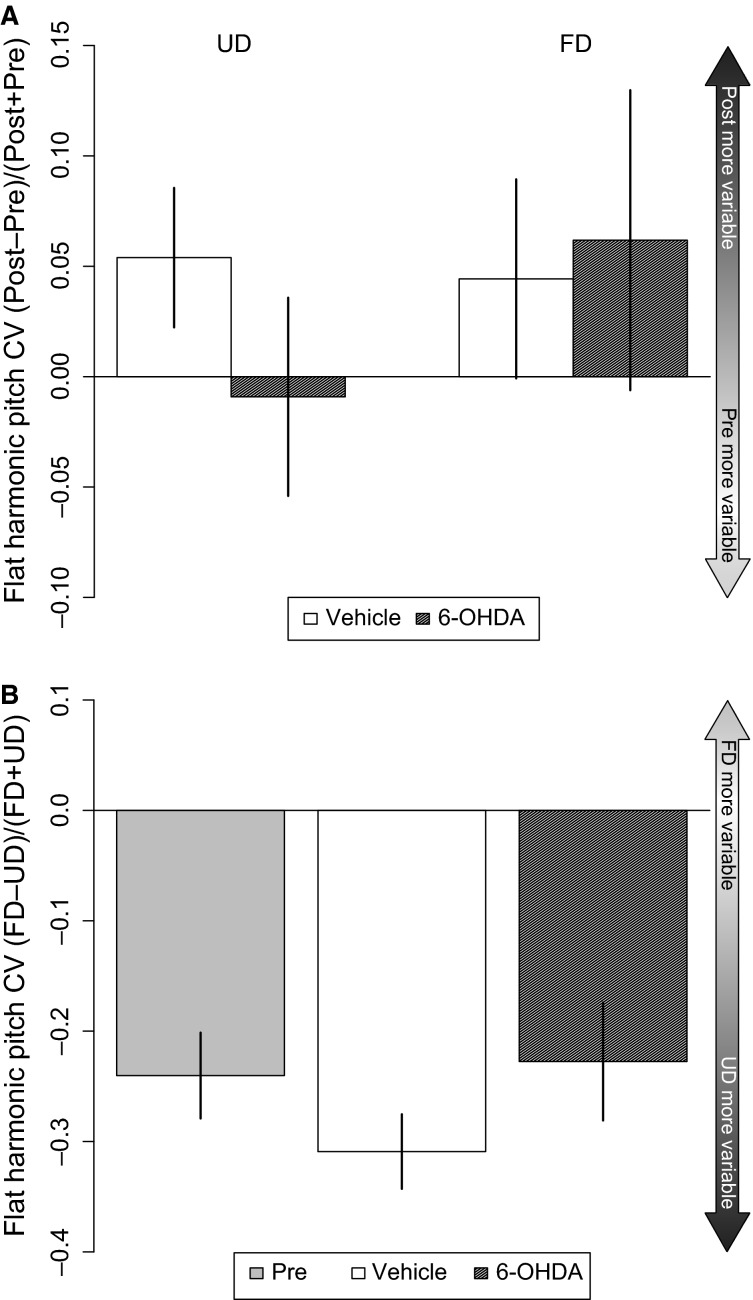
Social-context-dependent differences for harmonic syllables are not affected by 6-OHDA injection in Area X. A comparison of UD versus FD song features following vehicle or 6-OHDA injection was made using the effect size plots with some refinement: The “Pre” bar represents all pre-surgery syllables combined from both pre-treatment groups. The “vehicle” bar represents comparisons between post-vehicle FD and post-vehicle UD whereas the “6-OHDA” bars are comparisons between post-6-OHDA FD versus post-6-OHDA UD song. (A) No significant difference was observed between vehicle and 6-OHDA on flat harmonic syllable CV in UD (left) or FD (right) song. (B) Before surgery (“Pre”) UD song has greater pitch variability than FD song. Following surgery (“6-OHDA” and “Vehicle”), pitch variability continues to be greater in UD song.

Acoustic features in zebra finch song are differentially modulated depending on social context: Syllable subtypes known as harmonic stacks have higher variability in fundamental frequency (FF) reflected as higher CV scores in UD versus FD song (Kao et al. 2005). Comparison of the FF CVs for 28 syllables in our own data prior to vehicle or 6-OHDA injection is consistent with prior reports (Table1; Figure S2). These syllable subtypes which are modulated by endogenous DA (Leblois et al. 2010; Leblois and Perkel 2012), were not altered here by 6-OHDA; social-context-dependent differences persisted post-injection (paired-plots, Figure S3A–D; *n* = 15 vs. 13 syllables). Effect size plots also indicate no significant effect of 6-OHDA on these CV scores (Fig.6A) and the UD versus FD differences in FF variability were preserved (Fig.6B). However, a power analysis of these syllable subtypes revealed that the ability to detect differences due to the drug is only 10% (Table1).

**Figure 7:**
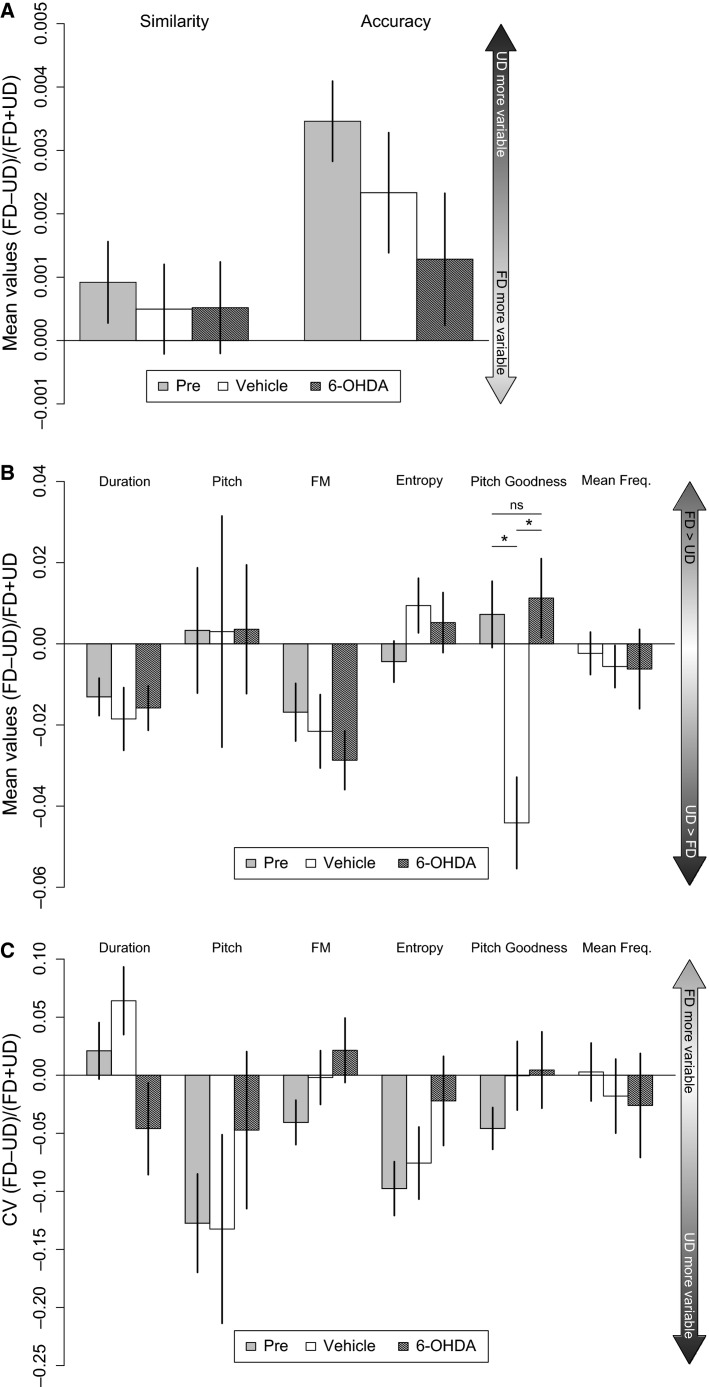
Social-context-dependent mean and CV scores for all syllable types are not affected by 6-OHDA injection in Area X. (A) Syllable self-similarity and self-accuracy are greater in FD than UD song before surgery (“Pre”; positive bars). Greater self-similarity and self-accuracy indicate less variability across multiple renditions of the same syllable. Neither injection with vehicle nor 6-OHDA caused a significant change in this trend. (B) The effect of injection with vehicle or 6-OHDA did not cause a significant change in mean scores between FD and UD songs except for pitch goodness in which UD became greater than FD in vehicle birds (*). (C) The effect of injection with vehicle or 6-OHDA did not cause a significant change in the modulation of variability (as measured by CV) between FD and UD songs. Positive bars indicate greater variability in FD while negative bars indicate greater variability in UD. In the pre-surgery group, the negative bars of greater magnitude for pitch and entropy indicate that these features were more variable for UD versus FD song.

An investigation of all syllable subtypes (harmonics included) revealed that pre-surgery, FD song had higher syllable self-similarity and accuracy scores compared to UD song (Fig.7A, “pre”). Pitch and entropy changes were more variable during UD versus FD song in the pre-surgery group (Fig.7C). Following 6-OHDA or vehicle injection, these differences were preserved. Overall, the mean and CV scores for song features indicated no attenuation of social-context-dependent differences with 6-OHDA (Figs.7B and C). Unexpectedly, one feature, mean pitch goodness (Fig7B), for the vehicle group was greater in UD than FD.

### 6-OHDA administration and non-song motor features

An observer blind to the treatment scored the frequency of non-song behaviors (eating, drinking, alarm calls, grooming, flying, beak-wiping, preening and following the female) in a subset of the vehicle (*n* = 4) and 6-OHDA injected birds pre versus post-injection for both UD and FD states (*n* = 6). Comparing the pre versus post-surgery vehicle group, no changes in behavior were detected. Following 6-OHDA injection, the only feature that changed was increased beak-wiping during FD (resampling paired difference, *P* < 0.05), but this behavior was rare, limited to 1–2 wipes/session.

## Discussion

Bilateral injection of a 1.2 *μ*g dose of 6-OHDA targeted to Area X reduced DA signal within this song control nucleus, as measured by quantification of TH levels on immunoblots. No changes in postsynaptic DARPP-32 levels within Area X were detected. The accuracy of our targeting was validated by the lack of change in TH and DARPP-32 signals in outlying VSP. A relatively novel aspect of our approach was to obtain tissue punches from both rodent and finch basal ganglia regions in order to quantitatively measure DA signals from entire nuclei via immunoblotting. This method can provide a more complete picture of overall DA loss allowing for multiple animals to be analyzed on one blot. A qualitative immunohistochemical image of TH fiber loss in Area X due to 6-OHDA injection (Fig.2C) supports the more quantitative results obtained from the immunoblots. Based on the unique aggregation of birdsong control neurons within their surrounding brain regions, this approach further offers the opportunity to manipulate DA levels specifically within a vocal control region of the basal ganglia.

Basal ganglia tissue and song measurements were sampled over an acute phase of treatment, 4–5 days following injection of 6-OHDA, in order to quantify the TH loss and subtle changes within the bird’s song over an early time window. This period was selected to model the effects of early DA loss on vocal symptoms due to the loss of TH-positive axons in the striatum prior to death of midbrain DA cells (Kirik et al. 1998; Morales et al. 2015). In rodent models of early disease, synaptic degeneration and loss of TH-positive axons in the striatum can be detected as early as 24 h post-injection and becomes more marked by 5 days. This loss of TH-positive axons precedes the retrograde degeneration and death of the midbrain DA cells that takes place over weeks to months (Morales et al. 2015).

The main differences following 6-OHDA injection in rat versus finch lie in the severity of the lesion and associated vocal symptoms. In the rodent literature, the striatal TH loss varies in severity depending upon the site of injection (lateral ventricles, median forebrain bundle) and the 6-OHDA dosage used, with higher doses resulting in a more complete loss of TH fibers (Morales et al. 2015; Przedborski et al. 1995; Grant et al. 2015). The associated degradation in the vocal signal affects some, but not all, features such as frequency-modulated complex calls (Ciucci et al. 2007; Ciucci et al. 2009). In contrast, here in zebra finch Area X, reduction in the DA biomarker TH results in subtle loss of UD song variability likely due to the lower 1.2 *μ*g dose of 6-OHDA. The higher 7 *μ*g dose used in rodents (Grant et al. 2015) proved lethal to finches, and doses >1.2 *μ*g can induce a lesion in Area X (data not shown).

We predicted that UD song would be more sensitive to 6-OHDA effects than FD song, given that DA levels in Area X are already quite low during UD singing (Sasaki et al. 2006); a small loss of DA would thus proportionally affect more of the UD than the FD-associated levels. Confirming this prediction, decreases in variability were detected in syllable accuracy scores (syllables became more similar across renditions) and reduced variability in mean frequency. Strong trends for decreased variability in individual features that comprise the accuracy score calculation were detected post-6-OHDA injection for syllable duration, frequency modulation, entropy, and pitch goodness (Table1) that were not detected in vehicle-injected birds. These trends were evident for both the effect size and the raw data.

The effect sizes (Figs.4–7; see Methods) of each treatment or social context on every syllable, provide a direct statistical comparison between conditions. In addition to effect size, Table1 presents the results of statistical tests where song data were paired, and analyses were performed within a condition. The two methods show overall agreement; in a few instances significance is observed when the data are viewed as pairs but not when calculating the effect size. While both results are valid, we focus on the effect size because this transformation enables the direct comparison between two conditions (e.g., vehicle vs. 6-OHDA); a comparison not possible when data are analyzed as pairs (e.g., pre-vehicle vs. post-vehicle). Additionally, calculating differences between raw values when data are paired allows large and small values to heavily skew the overall difference between conditions, which is alleviated by the normalization in calculating effect size. Plotting the effect size also clearly indicates the magnitude of the change resulting from the experimental manipulation, or condition.

Using these analyses, 6-OHDA-induced changes were detected in UD but not FD song. The reduction in UD song variability following DA-depletion is reminiscent of vocal changes in human PD in which reduced vocal variability is evident in a wide range of symptoms including breathy, soft, rough and monotonous voice, impairment in coordination of orofacial articulators, and fluency (reviewed in Sapir (2014). Symptoms such as monotonous voice occur early in the disease (Harel et al. 2004) before the large-scale loss of DA cells in the SNc. In contrast, because FD song is associated with elevated Area X DA levels (Sasaki et al. 2006), the DA loss associated with the 1.2 *μ*g dose may be proportionally too low to impact FD. Interestingly, the lack of detectable changes during FD song is reminiscent of the observation that PD patients perform better when externally cued, for example, by a speech-language pathologist (Ho et al. 1999). The apparent lack of 6-OHDA’s effect on FD may be a consequence of low power (see below) and/or reflect compensatory mechanisms related to external cues that override neuropathological deficits.

We next evaluated the effect of 6-OHDA induced reduction in DA signals in Area X on social context-dependent differences normally evident during UD versus FD song. Zebra finch FD song is characterized as being more stereotyped, based upon the analysis of one particular syllable type (Kao et al. 2005). Specifically, syllables with low frequency-modulation, known as harmonic stacks, have reduced variability in FF from rendition to rendition in FD song compared to UD (Kao et al. 2005). Pharmacological blockade using a D1 receptor antagonist in Area X causes these harmonic syllables to become more variable during FD song (Leblois and Perkel 2012). The higher variability in FF in UD versus FD song was observed here prior to 6-OHDA injection, replicating prior findings. Unexpectedly, there was no detectable effect of mild 6-OHDA-mediated DA depletion on variability scores. Power analysis indicated that the ability to detect any such effect was limited by the low number of harmonic stacks available to analyze. These make up only a subset of all syllable types unless birds are selectively bred to obtain multiple harmonic syllables in their motifs.

To date, one other study has examined natural differences in UD versus FD song at the syllable level, reporting that subsyllabic elements in the bird’s song are more spectrally similar in FD than UD (Leblois and Perkel 2012). Our acoustic analysis examined a wider range of song features between the two social contexts and found that pre-surgery, FD song has higher self-similarity and accuracy scores across multiple renditions, supporting the previous literature that FD song is more stereotyped compared to UD. Although 6-OHDA affected small changes in UD song features observed pre versus post-surgery (Fig.4A and C) it was not sufficient to cause UD song to fully resemble FD song.

Receptor-mediated mechanisms may underlie 6-OHDA effects on UD song. In rats, D1 and D2 receptor activation modulate their ultrasonic vocalizations both separately and synergistically (Ciucci et al. 2013). The function of these receptors in vocalizations may be differentially altered with 6-OHDA administration. For example, injection into the SNc of rodents results in elevated striatal D2 receptor mRNA levels, whereas D1 receptor mRNA is reduced (Qin et al. 1994). In adult male zebra finches, D2 receptors appear to be the prominent basal ganglia subtype (Kubikova et al. 2010). Yet, as mentioned, a D1 receptor antagonist causes FD song to resemble UD song (Leblois and Perkel 2012). Because many Area X MSNs co-express both D1 and D2 receptors (Kubikova et al. 2010), discerning the subtype specific effects of 6-OHDA injection will be challenging. Moreover, the traditional view that activation of D1 receptors promotes excitability in the direct pathway whereas D2 suppresses it in the indirect pathway has been revised to recognize that the mammalian striatopallidal pathways and projections are anatomically and physiologically intertwined (Calabresi et al. 2014; Kupchik et al. 2015).

Although it is likely that DA receptors are abnormally activated following 6-OHDA administration, altered adrenergic signaling cannot be excluded. DA can bind to alpha-2 type adrenergic receptors, which are abundant in Area X (Cornil et al. 2008). Ongoing work is aimed at assessing any alterations in these receptor levels following 6-OHDA administration. Norepinephrine (NE) from the locus coeruleus can also bind to adrenergic receptors, but there is sparse NE in Area X based on HPLC measurements (Gale and Perkel 2005) and immunostains for dopamine beta hydroxylase, the biosynthetic enzyme for NE (Castelino and Schmidt 2010).

The progressive neuropathology associated with PD in the brainstem and cortico-basal ganglia circuits (Braak et al. 2003) contributes to voice and speech symptoms, but the underlying neural mechanisms are not well-understood. The 6-OHDA zebra finch model described here provides a convenient entry point to examine the impact of mild DA loss on synaptic mechanisms and song. By studying song circuitry, inferences can be made regarding neural mechanisms underlying early vocal changes in human PD. Indeed, the special song-dedicated nucleus, Area X, shares more similar gene expression patterns with the putamen, a speech active region in humans than with these areas in non-vocal learning birds and primates (Pfenning et al. 2014). Future investigations will use 6-OHDA as a tool to identify nigrostriatal genes sensitive to DA depletion (Hilliard et al. 2012a; Hilliard et al. 2012b). Combining genetic, physiological, and behavioral approaches in the well-characterized vocal circuitry of the songbird will advance understanding of circuits that drive their vocal apparatus with implications for humans.

## Supporting Information

**Figure. S1.** Validation of syllable independence. (A) Acoustic similarity relationships between syllables from a zebra finch. A series of boxplots depicting similarity scores based on comparisons made between 25 renditions of syllable “A” with 25 renditions of each other syllable in the motif (i.e., 600 self-comparisons and 25 cross-syllable comparisons). Horizontal lines inside each box represent the median, box edges represent the 1st and 3rd quartiles, lines extending from the boxes represent the highest and lowest values of the dataset that are within 1.5 times the interquartile range, and data points extending beyond this range are depicted as open circles. (B) Resampling statistics to test for syllable independence. To determine whether variability in fundamental frequency differed across syllables, a resampling test was performed between the most variable and least variable syllable from one bird. The actual pitch scores from 25 renditions of each syllable were used to generate a CV and the difference between these CVs was used as a test statistic. The actual test statistic (in red) was compared to the distribution of resampled CV differences. This generated a *P*-value of 0.0026, which indicates a statistically significant variability between the two observed syllables. Therefore, all syllables were treated as independent of each other for the purposes of mean and CV analyses.

**Figure. S2.** Paired plots of pre-surgery undirected (UD) and female-directed (FD) harmonic syllable scores. Each circle represents an individual syllable. Higher fundamental frequency variability (FF CV) is detected during UD but not FD song for these specific syllable types (resampling paired mean difference, *P* < 0.0001). Individual syllables are presented in gray and the group mean is overlaid in black.

**Figure. S3.** Paired plots of pre versus post-surgery UD and FD harmonic syllable scores with 6-OHDA or vehicle injection. Individual syllables are presented in gray and the group mean is overlaid in black. (A) Paired plots for harmonic syllables pre versus post-6-OHDA injection into Area X reveal no significant differences for FF CV during UD song. The bold face line is the mean. (B) Paired plots for harmonic syllables pre versus post-vehicle injection into Area X reveal no significant differences for FF CV during UD song. The bold face line is the mean. (C) Paired plots for harmonic syllables pre versus post-6-OHDA injection into Area X reveal no significant differences for FF CV during FD song. The bold face line is the mean. (D) Paired plots for harmonic syllables pre versus post-vehicle injection into Area X reveal no significant differences for FF CV during FD song. The bold face line is the mean.
