# Supplementary figures and images for: Reduced vocal variability in a zebra finch model of dopamine depletion: implications for Parkinson disease
Source: Physiol Rep. 2015 Nov 12;3(11):e12599. doi: 10.14814/phy2.12599 (PMC4673629; doi:10.14814/phy2.12599)

Supplemental Figure 1A

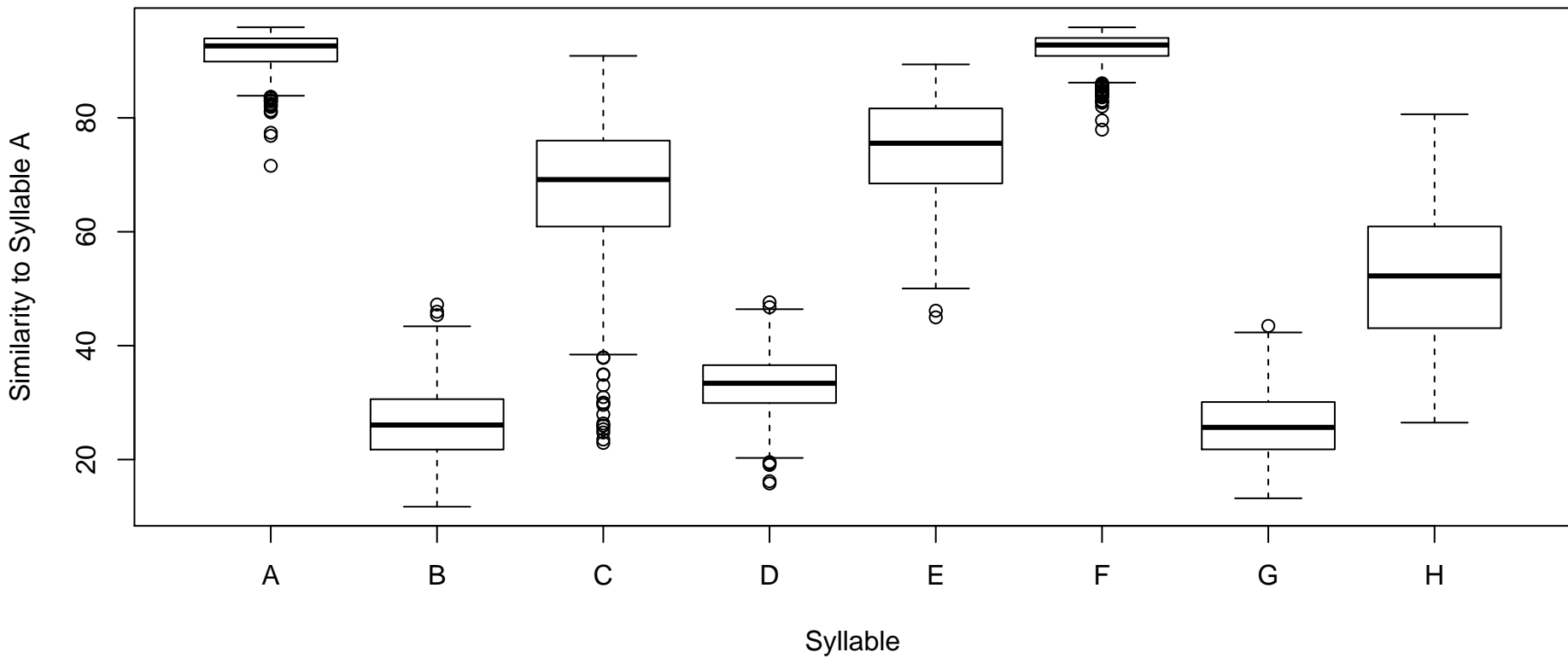

Supplement: Supplementary file 1 [file phy20003-e12599-sd1.pdf]

Supplemental Figure 2

**Pre-surgery**

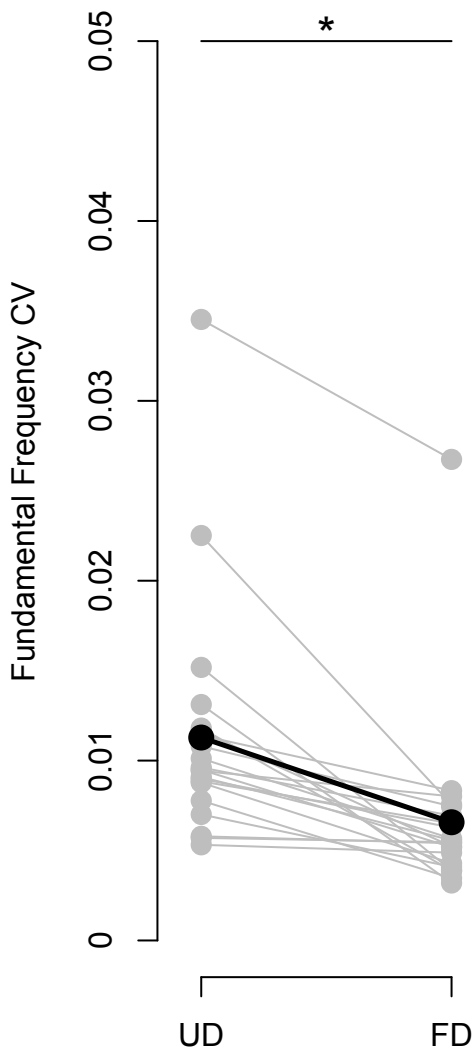

Supplement: Supplementary file 2 [file phy20003-e12599-sd2.pdf]

Supplemental Figure 3A

**UD 6-OHDA**

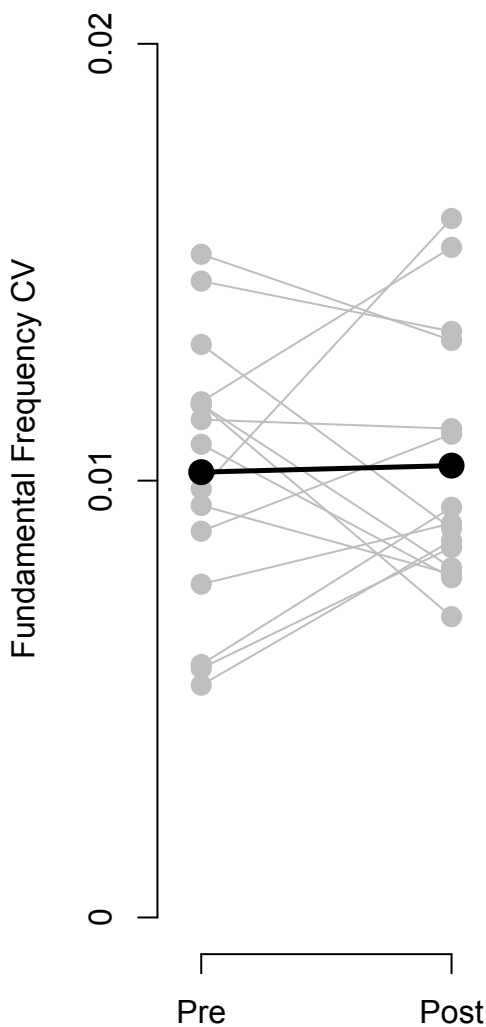

Supplement: Supplementary file 3 [file phy20003-e12599-sd3.pdf]

Supplemental Figure 1B

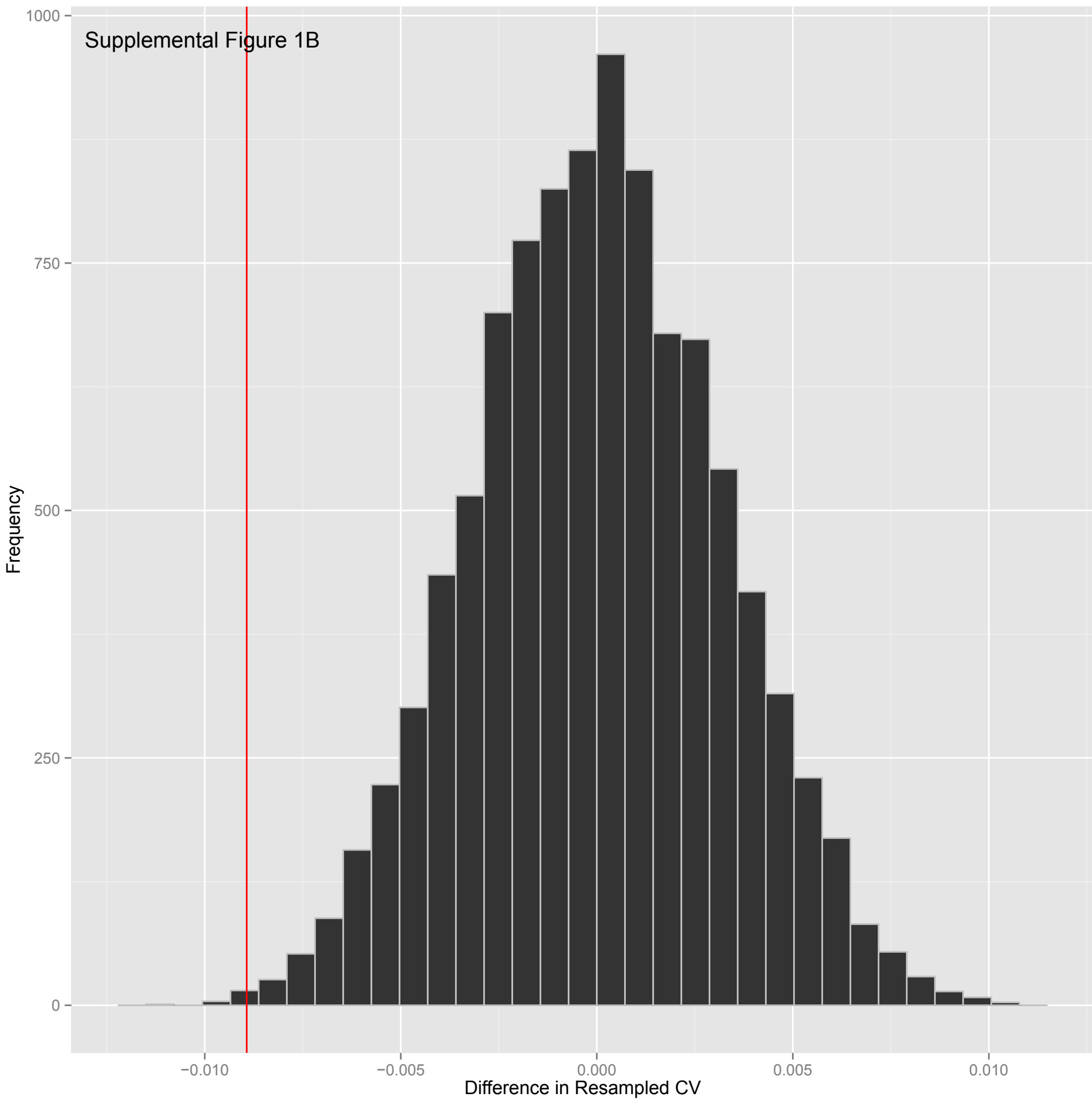

Supplement: Supplementary file 4 [file phy20003-e12599-sd4.pdf]

Supplemental Figure 3B

# UD Vehicle

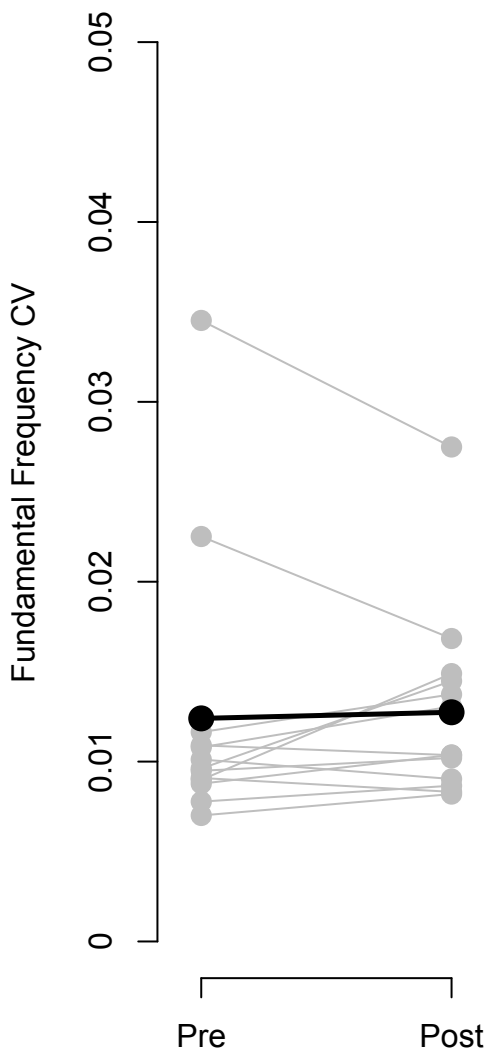

Supplement: Supplementary file 5 [file phy20003-e12599-sd5.pdf]

Supplemental Figure 3C

**FD 6-OHDA**

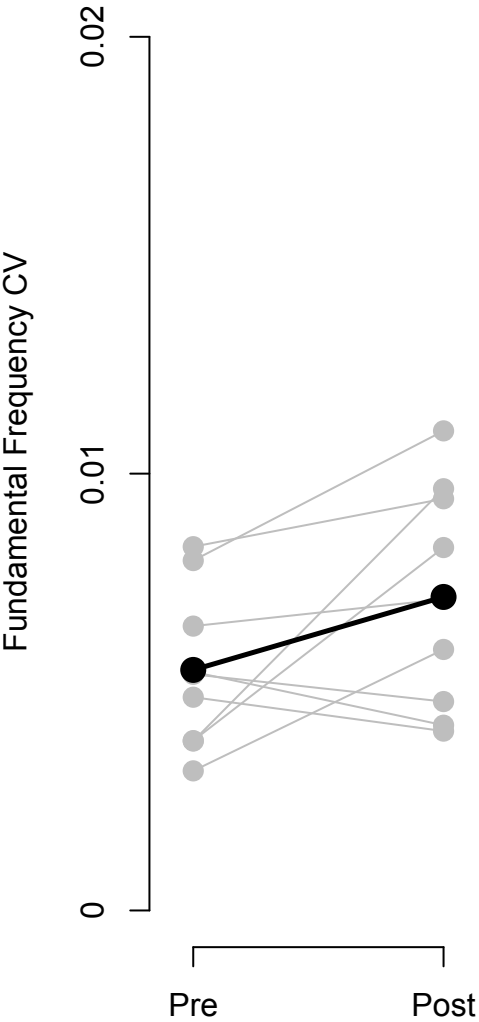

Supplement: Supplementary file 6 [file phy20003-e12599-sd6.pdf]

Supplemental Figure 3D

**FD Vehicle**

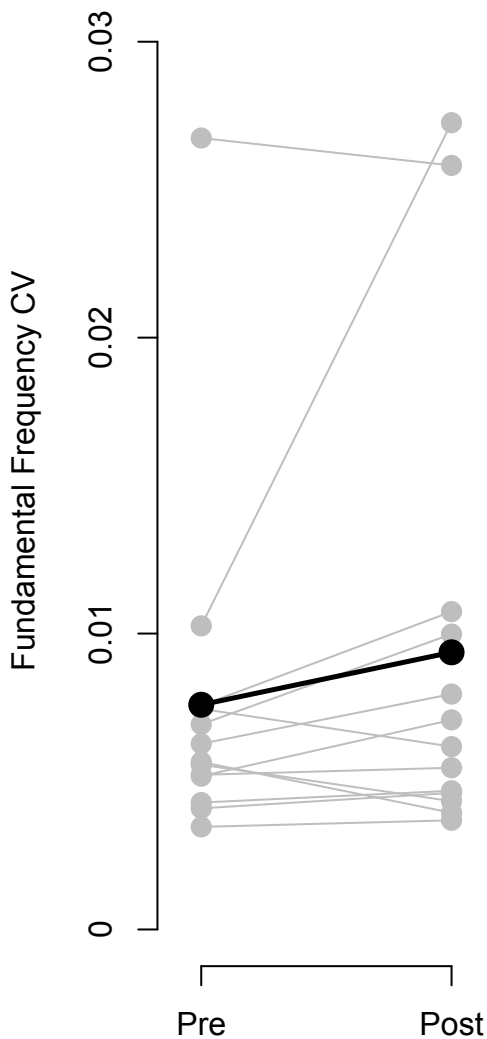

Supplement: Supplementary file 7 [file phy20003-e12599-sd7.pdf]
