# Supplementary material for: Reduced vocal variability in a zebra finch model of dopamine depletion: implications for Parkinson disease
Source: Physiol Rep. 2015 Nov 12;3(11):e12599. doi: 10.14814/phy2.12599 (PMC4673629; doi:10.14814/phy2.12599)
Supplement: Supplementary file 8 [file phy20003-e12599-sd8.docx]

Supplementary Statistics

*Syllable independence*

To determine whether syllables within a bird should be evaluated independently, or collapsed within one bird, SAP was utilized to compute similarity scores between groups of syllables in a pre-surgery bird. 25 renditions of one syllable were scored against themselves as well as 7 other syllables in the bird’s repertoire. This generated 600 self- comparisons and 625 cross-syllable comparisons for each of the 7 additional syllables **(Supplemental Figure 1A)**. When a syllable is more similar to itself than other syllables, it is considered independent. Therefore a one-way analysis of variance (ANOVA) was performed on the similarity scores and a p-value of p < 10^-4^ was achieved. This indicates that the syllables are independent of one another and therefore syllables were not collapsed within birds for the purpose of mean features and similarity scores. However, when multiple renditions were present in a motif, only the first occurrence of that syllable within each motif was used. This was done in order to prevent placing unequal emphasis on one unique element of a bird’s repertoire.

*Independence of syllable fundamental frequency variability*

To determine whether variability in fundamental frequency differed across syllables, a resampling test was performed between the most variable and least variable syllable from one bird. The actual pitch scores from 25 renditions of each syllable were used to generate a CV and the difference between these CVs was used as a test statistic. Due to the large difference in mean pitch for these syllables, actual pitch scores were ‘de- meaned’ by the group mean for each syllable. This allows preservation of variance while setting the means to be equal across these syllables. The de-meaned observations were combined into a resampling pool and groups equivalent to the number of original observations were drawn from this pool with replacement. CVs were calculated for each resampled group and the difference in CV was calculated and stored. This was repeated 10,000 times to generate a distribution of CV differences. The actual test statistic was then compared to this distribution to generate a p-value of 0.0026, which indicates a statistically significant variability between these two syllables was observed **(Supplemental Figure 1B)**. Therefore, for the purposes of variability analyses, all syllables were treated as independent of each other. Because significant differences in variability were observed for one syllable feature, all syllables were treated independently for all other acoustic feature variability analyses.
